# Risk Factors for Refractory and Delayed *De novo* Otitis Media Requiring Pressure Equalization Tube Insertion

**DOI:** 10.13188/2380-0569.1000008

**Published:** 2015-08-22

**Authors:** Sarah N. Bowe, Kris R. Jatana, D. Richard Kang

**Affiliations:** 1 Department of Otolaryngology, San Antonio Uniformed Services Health Education Consortium, JBSA Ft. Sam Houston, TX, USA; 2 Department of Otolaryngology, Nationwide Children’s Hospital and Wexner Medical Center at Ohio State University, Columbus, OH, USA; 3 Department of Otolaryngology, Boys Town National Research Hospital, Omaha, NE, USA

**Keywords:** Otitis media, Risk factors, Children, Pressure equalization tube insertion

## Abstract

**Objective:**

Limited data exists regarding risk factors for otitis media in older children and specifically those for which surgical intervention is performed. This study investigated potential risk factors in this older age group who required pressure equalization tube (PET) insertion.

**Study design:**

Retrospective cohort study

**Setting:**

Tertiary care pediatric academic medical center

**Subjects and methods:**

Children 6–12 years old undergoing PET insertion between October 1, 2010 and September 30, 2011. Data was stratified into two separate age cohorts (6–7 versus 8–12-year-olds) and compared using chi-square analysis.

**Results:**

A total of 263 patients met study criteria. PET insertion was most common in 6 year-olds (36%, 95/263). Presence of siblings (p=0.03) and history of recurrent upper respiratory tract infection (p<0.01), otalgia (p<0.05), otorrhea (p<0.001), and nasal discharge (p<0.001) were common in the older cohort. No statistical difference was found for history of recurrent acute otitis media, allergy, asthma, or atopy between the two groups (p=0.23–0.92), although the overall prevalence of these conditions was high in both cohorts.

**Conclusion:**

The 8–12-year-olds had a history of recurrent upper respiratory tract infection and more infectious symptoms than the 6–7-year-olds. Atopy can lead to a heightened susceptibility to upper respiratory tract infections and potential increase in the relative risk of otitis media. In our patient population, while there was no statistically significant difference in history of asthma, allergy, or atopy, the overall prevalence within both cohorts was relatively high. Therefore, this study provides insight into many pertinent and potentially modifiable risk factors for older children requiring PET insertion.

## Introduction

Chronic otitis media (COM) and recurrent otitis media (ROM) are two of the most common infectious diseases worldwide. COM and ROM affect diverse cultural and racial groups distributed in both developing and industrialized countries. The risks of ROM or COM include suppurative complications, antibiotic resistance, tympanosclerosis, retraction pockets, ossicular chain erosion, cholesteatoma, and conductive hearing loss that might affect language, speech, or psychological development. Tympanostomy tube insertion is the main surgical intervention for otitis media. Each year in the United States, 667,000 children under 15 years old receive tympanostomy tubes, which accounts for more than 20% of all ambulatory surgery in this age group [1]. At an average cost of $2700 per procedure, this contributes approximately $1.8 billion in health care costs and doesn’t include the added financial burden from follow-up care, treatment of otorrhea, and management of other complications [1].

Otitis media with effusion has a bimodal distribution with a prevalence rate of 20% in the first and largest peak at age 2 and 16% with the second peak around 5 years of age [2]. The immaturity of function of both the immune system and the eustachian tube are considered the main responsible factors for the development of otitis media. Unfortunately, there is a subset of children that continue to suffer from ROM or COM after age 5. Effective treatment for these individuals depends on a thorough understanding of pertinent risk factors.

Multiple cross-sectional, cohort, and case-control studies have examined the risk factors of COM/ROM, including demographic, environmental, and patient-related factors. The majority of these studies have been performed on younger patients, in foreign countries, and using various diagnostic criteria for COM or ROM. Limited data exists in older children and, more importantly, specifically those for which surgical intervention is performed. This study investigated risk factors for *de novo* or refractory otitis media in 6–12 year old patients who required new or recurrent pressure equalization tube (PET) insertion. In particular, we sought to compare our older age cohort (8–12 year-olds) to our younger age cohort (6–7 year-olds) to characterize risk factors that may help clinicians distinguish children with a poor natural course (e.g. those that would benefit from close follow-up or earlier intervention) from those with a more favorable course. We expect that older children will have a higher prevalence of known risk factors, such as allergy, asthma, and atopy, compared to their younger counterparts.

## Methods

An extensive literature review was conducted to identify risk factors for COM/ROM with a particular focus on those studies in school-age children [3–12]. Based on this information, a data collection form with pertinent factors relating to demographics, social environment, and patient history was developed. The Research Institute at Nationwide Children’s Hospital deemed the study eligible for IRB exempt review.

A database query for children aged 6–12 years-old undergoing tympanostomy tube insertion (CPT 69436) at Nationwide Children’s Hospital, a tertiary care pediatric hospital, was performed between October 1, 2010 and September 30, 2011. Three hundred and forty six patients met the inclusion criteria, namely PET insertion during the stated time interval. Children with a history of craniofacial or syndromic abnormalities (e.g. Cleft palate or Down syndrome), immunodeficiency syndrome (e.g. Ig A deficiency), and chronic diseases requiring frequent prophylactic or therapeutic antibiotics (e.g. Cystic Fibrosis) were excluded from the study. The medical records were reviewed and two hundred and sixty three patients met the necessary criteria for inclusion.

Data was obtained for a younger age cohort (i.e. 6–7 year-olds) and an older age cohort (i.e. 8–12 year-olds). Statistical analysis was carried out to identify possible risk factors for PET insertion in the older pediatric population. Chi-square tests were used to assess differences between a risk factor and presence in the younger vs. older cohort. Odds ratio and 95% confidence intervals were also calculated. A p-value < 0.05 was considered statistically significant.

## Results

**Figure 1:**
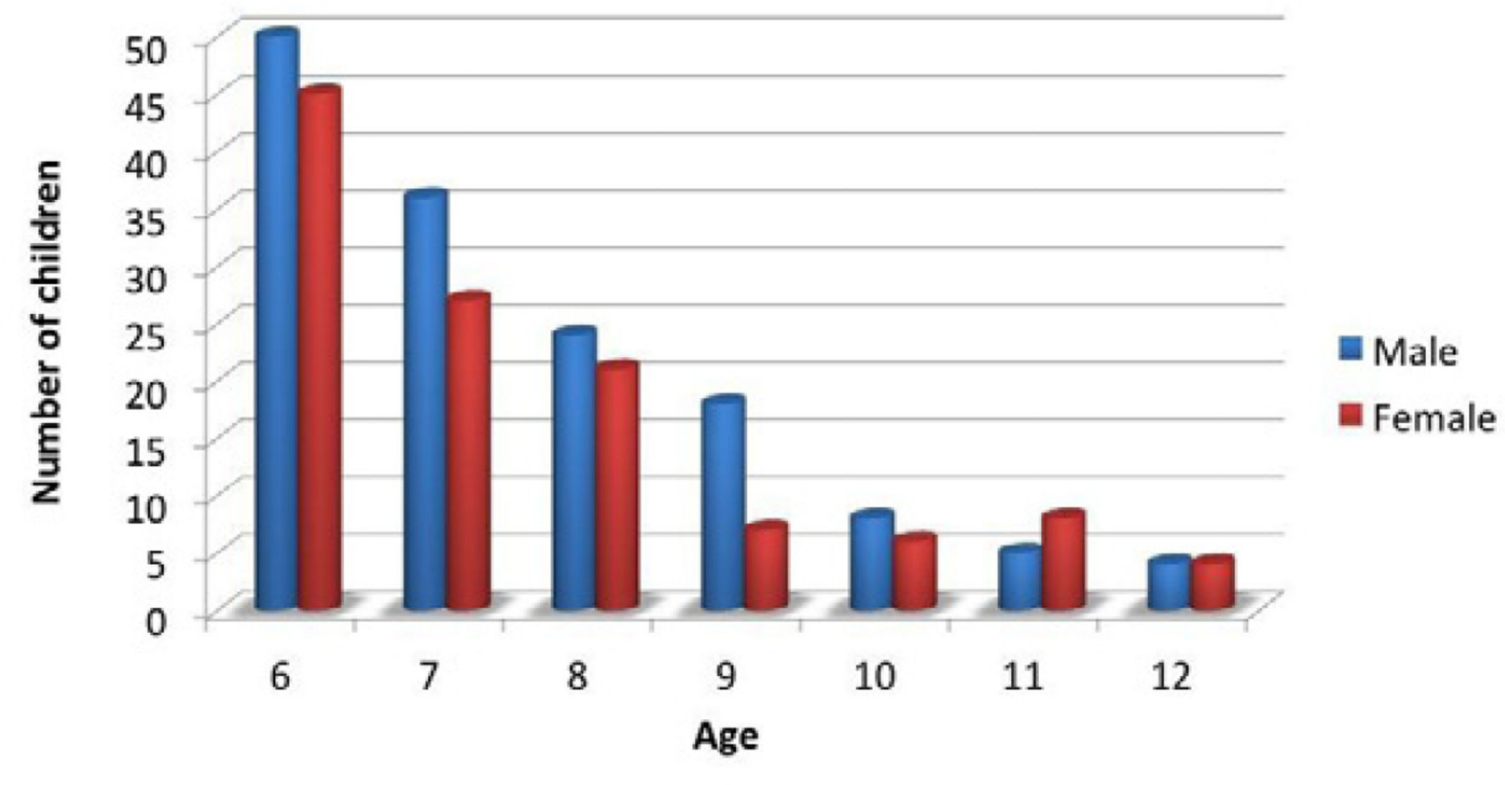
Number of children undergoing PET insertion according to age.

Two hundred and sixty-three medical records were included in this study. In the selected population of children between the ages of 6 and 12 years old undergoing new or recurrent PET insertion, the mean age was 7.5 years old. There were 145 males (55.1%) and 118 females (44.9%) in total. Overall, males were found to undergo tympanostomy tube insertion slightly more than females (55.1, 95% CI 49.1–61.0 vs. 44.9, 95% CI 39.0–50.9, respectively). PET insertion was most common amongst 6 year-olds (36.1%, 95/263), which decreased by age (Figure 1).

Table 1 demonstrates the comparison of risk factors between the younger age cohorts vs. the older age cohort. No significant gender-based differences were observed (p = 0.78). The presence of siblings was found was to be higher within our older group of children (OR 1.72, p =0.03). History of recurrent upper respiratory tract infections (OR 3.6, p = 0.005), as well as symptoms of otalgia (OR 1.97, p = 0.01), otorrhea (OR 7.26, p < 0.0001), and nasal discharge (OR 2.46, p = 0.0005) were also prominent in the older cohort. There was a trend for history of hearing loss (OR 1.97) and presence of parental smoking (OR 1.61) among 8–12 year-olds, although the results did not reach statistical significance (p = 0.06–0.09).

There was a history of snoring (OR 0.54, p = 0.02) and recurrent tonsillitis (OR 0.3, p = 0.0003) that was more common in the 6–7 year-old children. Previous otolaryngologic surgery was more frequent in the older group (OR 4.3, p = < 0.0001). Otherwise, there was no significant difference (p = 0.23–0.92) identified when comparing history of recurrent acute otitis media (OR 0.98), allergy (OR 0.73), asthma (0.92), or atopy (OR 0.87) between the two groups.

## Discussion

Chronic otitis media and recurrent otitis media are common health problems, accounting for significant health care utilization. In addition, these conditions may result in more serious otologic complications, as well as hearing loss with an effect on speech and language development. A thorough understanding of etiologic factors for COM/ROM can aid physicians in recognizing unfavorable conditions that can prompt earlier specialist care or medical or surgical intervention. More importantly, if modifiable risk factors are identified, then measures can be taken to prevent and decrease the onset of disease.

Age is one of the most important risk factors for otitis media and, as a result, only a small subset of children will require PET insertion after age 6 [2–4,11]. In the present study, tympanostomy tube insertion was most common amongst 6 year-olds (36.1%, 95/263) and decreased substantially with increasing age, such that only 3% of PET insertions were performed on 12 year-olds (8/263) (Figure 1).

The influence of gender is controversial with some studies showing a higher prevalence in males [10,11], other studies in females [7], and the remainder failing to show an effect of gender on otitis media [4,5,8]. Our results showed that males were found to undergo tympanostomy tube insertion slightly more than females (55.1, 95% CI 49.1–61.0 vs. 44.9, 95% CI 39.0–50.9, respectively). This observation was not found to differ significantly when the younger cohort was compared to the older cohort (p = 0.78).

The impact of family size and/or number of siblings on otitis media has also varied within the literature. A few studies have shown a statistically significant influence on the presence of otitis media [2,4], while others have failed to identify a relationship [5,11]. The likelihood that a child in the 8–12 year-old cohort had siblings in the home was increased compared to those in the younger group (OR 1.72, p = 0.03).

Upper respiratory tract infections (URTIs) have been universally identified as a significant prognostic factor for COM/ROM [5–8]. Studies have shown that the opening and closing function of the eustachian tube (ET) [13], as well as the extent of inflammation on ET mucosa [14] are affected by URTIs. Within our older cohort, a history of recurrent upper respiratory tract infections (OR 3.6, p = 0.005), as well as symptoms of otalgia (OR 1.97, p = 0.01), otorrhea (OR 7.26, p < 0.0001), and nasal discharge (OR 2.46, p = 0.0005) was more prominent than within our younger patients. As a result, prevention of URTI is an important step to help decrease the prevalence of otitis media.

Identification of hearing loss within the patient’s history has generally not been a reliable measure for presence of otitis media. Caylan et al. evaluated both parental and teacher awareness of hearing loss in patients and found a positive predictive value of only 11.7% and 7.5%, respectively, for the presence of otitis media with effusion (OME) [11]. In contrast, both parents and teachers were able to provide a history of hearing loss in more patients with OME (30–38.9%) compared to those without (10.2–10.6%) [11]. Our results showed a trend for increased history of hearing loss within the 8–12 year-old cohort (OR 1.97, p = 0.06). In this study, there was a history of hearing loss identified in 79.7% of 6–7 year-olds and 88.6% of 8–12 year-olds. The high prevalence noted in our study, in comparison to Caylan et al., is likely due to the fact that OME can present in an insidious manner such that children, parents, and/or teachers may not identify a concern regarding hearing, especially if unilateral. In our patient population that has undergone PET insertion, most patients have undergone formal hearing testing and therefore presence of hearing loss has been confirmed, which would be noted in the history.

Exposure to smoking has been one of the most studied risk factors for OME. Similar to gender, the literature has shown mixed results. Some studies have failed to indicate any association with parental smoking and otitis media [6,11,12,15], while others demonstrate a clear relationship [4,5,8]. There was a trend toward increased parental smoking within our older patient cohort compared to our younger cohort (OR 1.61, p = 0.09). Perhaps more interesting is examining the rate of parental smoking exposure. Compiling rates from the previous literature, approximately 14.2% of those with OM had exposure to passive smoke [4,6–8,11,12]. In contrast, our patients who have undergone PET insertion for COM/ROM had exposure rates of 22.2–31.4%. While we are unable to statistically evaluate this information, it certainly exposes a potentially more pertinent risk factor amongst older children requiring tympanostomy tube insertion.

Both recurrent tonsillitis (OR 0.3, p = 0.0003) and history of snoring (OR 0.54, p = 0.02) were more common in 6–7 year-old children. On the other hand, otolaryngologic surgery was noted to be more prominent in the older group (OR 4.3, p = < 0.0001). Within our patient population, this likely represents the fact that these children have been tied in to the healthcare network and those younger patients with history of recurrent tonsillitis and/or snoring would be considered as candidates for tonsillectomy and adenoidectomy in conjunction with any tympanostomy tube insertion procedures. The close relationship between snoring and otitis media has been confirmed across numerous studies [4–7,11]. While it is important to note that this risk decreased significantly between the 6–7 year-olds and 8–12 year-olds, the overall rate of snoring within our older cohort is still relatively high (47.6%). Habitual snoring, defined as the presence of loud snoring at least three times per week, is epidemiologically linked to many otitis media risk factors and has been found to affect up to 27% of children [4,7]. This lends support to the clinical practice guideline on OME that recommends that physicians focus on suspected conditions (e.g. snoring) that may be associated with or may contribute to OME exacerbation [16]. As a result, we should consider the presence of snoring as a modifiable risk factor that may be addressed early on in these patients requiring new or recurrent PET insertion.

There was no significant difference (p = 0.23–0.92) identified when comparing history of recurrent acute otitis media (OR 0.98, 95% CI 0.57–1.69), asthma (OR 0.92, 95% CI 0.51–1.66), allergy (OR 0.73, 95% CI 0.44–1.21), or atopy (OR 0.87, 95% CI 0.33–2.28) between the two cohorts. While no difference was identified, it is important to examine the overall rates of these risk factors in both subgroups as it still provides further insight into modifiable factors that may reduce the prevalence of COM/ROM and need for PET insertion.

As one would expect, children who continue to experience episodes of acute otitis media (AOM) have an increased risk of developing chronic and recurrent middle ear infections. Multiple studies have shown that patients with a history of AOM significantly increase the risk of COM/ROM [5,7,11,12]. A history of recurrent AOM was present in 70.7% of our patient population that required PET insertion. Certainly, this high percentage indicates that the majority of these children possess factors that predispose them to continued acute infections (e.g. increased host susceptibility in the respiratory tract).

Relatively few studies have specifically evaluated the association between asthma and OME. Marchica et al. studied children over the age of 6 that required tympanostomy tube insertion and compared risk factors for recurrent placement [3]. They showed an increased prevalence of asthma (39%) in their total population and identified that almost half of those requiring recurrent tube insertion had asthma (49.4%). This was compared to a 13% prevalence of asthma in their geographical area. In our study, a history of asthma was relatively similar between the younger cohort (23.4%) and older cohort (21.9%). We do not have any local data with which to compare our rate, but identifying a greater than 20% prevalence of asthma in our overall population is pertinent for two reasons. First, it correlates with many studies that have identified the *Unified Airway Concept*, such that the inflammatory substrate found in the effusions of otitis media can be similar to the late-phase allergic response seen elsewhere in the respiratory tract, such as in allergic rhinitis and asthma [17,18]. Second, there has also been evidence of abnormal exercise-induced bronchoconstriction and elevated exhaled nitric oxide concentration, markers of bronchial hyper-reactivity and airway inflammation respectively, in children with ROM [19]. These findings should prompt a strong consideration for the presence of both allergy and asthma in any patients over 6 years old continuing to suffer from COM/ROM and requiring PET insertion.

As noted above, allergy or atopy appears to have a substantial association with COM/ROM [17,18]. Numerous studies continue to elucidate the strong link between allergy and otitis media with effusion [5,20–22]. In a study by Martines et al., 62.9% of their subject population (5–14 year-olds) with OME was found to have atopy on the basis of positive skin testing [7]. They found that the atopy percentage increased progressively with age, reaching a maximum percentage of 66.66% amongst 13–14 year-olds. They also examined the association between URTI and atopy and found that the relative risk for OME increases by 271%. Researchers have shown that atopic subjects tend to have a Th2-polarization and thus reduced Th1-response, placing these individuals at higher susceptibility to contract respiratory infections than non-allergic subjects [23,24]. Atopy not only predisposes individuals to higher rates of URTI, but also appears to have a compounding effect on risk for OME. In our patient population, there was no statistically significant difference in history of allergy between our younger cohort (43.7%) and older cohort (36.2%). The prevalence within our study is clearly less than that of Martines et al. [7]. While there can be environmental and population differences, perhaps this addresses a failure to evaluate for the presence of allergy/atopy, and certainly a modifiable risk factor if present.

Of course, as with all data collection based on chart reviews, our study suffers from several limitations. First, the presence or absence of risk factors may not have been appropriately or accurately recorded in the medical record. Ideally, every pertinent risk factor would have been addressed in every encounter, although this is not routinely the case. Likewise, there is an extensive wealth of information within the medical record and so pertinent risk factors may have been noted in an uncommon location and not identified by those performing the chart review. Third, while discussion regarding the manner to record data was performed prior to proceeding with the study, the input of the data may still vary amongst the given researchers. Finally, there are the inherent limitations of a retrospective analysis, such that we can identify prevalence and association, but not causation. Certainly, this study addresses numerous potential risk factors specifically within this population of older children requiring new or recurrent PET insertion, which provides a direction for future prospective studies.

## Conclusion

Otitis media is a common and multifactorial disease. While there is a substantial amount of knowledge regarding its risk factors, limited data exists in older children and, more importantly, those in which surgical intervention is performed. Of interest, within our older cohort, a history of recurrent upper respiratory tract infections, as well as symptoms of otalgia, otorrhea, and nasal discharge was more frequent than within our younger patients. Atopy has been associated with a heightened susceptibility to upper respiratory tract infections and, when both conditions are present, a substantial increase in the relative risk of otitis media. In our patient population, while there was no statistically significant difference in history of asthma, allergy, or atopy between our younger and older cohort, the overall prevalence within both was relatively high. Therefore, this study provides insight into many pertinent and potentially modifiable risk factors for older children requiring PET insertion.

## Figures and Tables

**Table 1:** Analysis of risk factors for PET insertion: Older vs. younger cohorts

Risk factors	6–7 year-olds n (%); n=158		8–12 year-olds n (%); n=105		Odds ratio	95% CI	p value
*Gender*							
Male	86	54.4	59	56.2	1.07	0.77–1.41	0.78
Female	72	45.6	46	43.8			
*Parental smoking*							
Yes	35	22.2	33	31.4	1.61	0.96–1.78	0.09
No	123	77.8	72	68.6			
*Presence of siblings*							
Yes	63	39.9	56	53.3	1.72	1.05–2.84	0.03
No	95	60.1	49	46.7			
*Snoring*							
Yes	99	62.7	50	47.6	0.54	0.32–0.89	0.02
No	59	37.3	55	52.4			
*Otalgia*							
Yes	94	59.5	78	74.3	1.97	1.14–3.38	0.01
No	64	40.5	27	25.7			
*Otorrhea*							
Yes	33	20.9	69	65.7	7.26	4.16–12.66	<0.0001
No	125	79.1	36	34.3			
*Hearing loss*							
Yes	126	79.7	93	88.6	1.97	0.96–4.03	0.06
No	32	20.3	12	11.4			
*Nasal discharge*							
Yes	45	28.5	52	49.5	2.46	1.47–4.12	0.0005
No	113	71.5	53	50.5			
*Nasal obstruction*							
Yes	32	20.3	25	23.8	1.23	0.68–2.28	0.49
No	126	79.7	80	76.2			
*Recurrent URTI*							
Yes	7	4.4	15	14.3	3.6	1.41–9.15	0.005
No	151	95.6	90	85.7			
*Recurrent tonsillitis*							
Yes	48	30.4	12	11.4	0.3	0.14–0.58	0.0003
No	110	69.6	93	88.6			
*Recurrent AOM*							
Yes	112	70.9	74	70.5	0.98	0.57–1.69	0.92
No	46	29.1	31	29.5			
*Positive allergy*							
Yes	69	43.7	38	36.2	0.73	0.44–1.21	0.23
No	89	56.3	67	63.8			
*Positive asthma*							
Yes	37	23.4	23	21.9	0.92	0.51–1.66	0.78
No	121	76.6	82	78.1			
*Positive atopy*							
Yes	12	7.6	7	6.7	0.87	0.33–2.28	0.78
No	146	92.4	98	93.3			
*Previous ENT surgery*							
Yes	81	51.3	86	81.9	4.3	2.39–7.73	<0.0001
No	77	48.7	19	18.1			

URTI: Upper Respiratory Tract Infection; AOM: Acute Otitis Media; ENT: Ear, Nose and Throat
